# Zinc Hyperaccumulation in Plants: A Review

**DOI:** 10.3390/plants9050562

**Published:** 2020-04-29

**Authors:** Habiba Balafrej, Didier Bogusz, Zine-El Abidine Triqui, Abdelkarim Guedira, Najib Bendaou, Abdelaziz Smouni, Mouna Fahr

**Affiliations:** 1 Laboratoire de Biotechnologie et Physiologie Végétales, Centre de biotechnologie végétale et microbienne biodiversité et environnement, Faculté des Sciences, Université Mohammed V de Rabat, 10000 Rabat, Maroc; 2 Equipe Rhizogenèse, Institut de Recherche pour le Développement, Unité Mixte de Recherche Diversité Adaptation et développement des Plantes, Université Montpellier 2, 34394 Montpellier, France

**Keywords:** zinc, hyperaccumulation, bioavailability, tolerance, molecular mechanisms, plants

## Abstract

Zinc is an essential microelement involved in many aspects of plant growth and development. Abnormal zinc amounts, mostly due to human activities, can be toxic to flora, fauna, and humans. In plants, excess zinc causes morphological, biochemical, and physiological disorders. Some plants have the ability to resist and even accumulate zinc in their tissues. To date, 28 plant species have been described as zinc hyperaccumulators. These plants display several morphological, physiological, and biochemical adaptations resulting from the activation of molecular Zn hyperaccumulation mechanisms. These adaptations can be varied between species and within populations. In this review, we describe the physiological and biochemical as well as molecular mechanisms involved in zinc hyperaccumulation in plants.

## 1. Introduction

Zinc (Zn) is the second most abundant transition metal in living organisms after iron [1]. Zn plays important roles in plant development, reproduction, and signaling due to its structural, catalytic, and activating functions [2]. Zn acts also as a cofactor for many enzymes, such as carbonic anhydrase, carboxypeptidase, and Zn-superoxide dismutase [3,4,5].

For most crops, the typical Zn concentration required for proper growth is approximately between 15 and 20 mg kg^−1^ dry weight [6]. Beyond these concentrations, Zn can be toxic to flora, fauna, and humans [7]. In plants, Zn toxicity increases with its bioavailability. The latter depends on different factors, such as pH, root exudate, microbial communities, and soil organic matter, that limit or promote its bioavailability [8,9,10]. Excess Zn may alter plant development [11,12]. However, some plants have developed the ability to grow in environments with high Zn concentrations where sensitive ones cannot [13]. Some of these plants, known as hyperaccumulators, have the ability to accumulate high amounts of Zn in their aerial parts [11,12,13]. To understand Zn hyperaccumulation mechanisms, at physiological, biochemical, and molecular level, scientists have firstly identified model plants as Zn hyperaccumulators [14,15]. To date, only 28 species have been described as Zn hyperaccumulators. 

In this review, we discussed the different sources of Zn contamination and parameters that can affect its bioavailability. We also reviewed the different studies underlying the effect of Zn excess on plant growth and development. Morphological, physiological, biochemical, and molecular adaptations to Zn in hyperaccumulator plants were also tackled in this review.

## 2. Sources and Bioavailability of Zinc

Zn occur naturally in soil from pedogenetic processes of mother rocks’ leaching [16]. Natural background levels of Zn in water, soil, and rocks change over a wide range of concentrations. Thus, its background in soils and rocks is typically between 10 and 300 ppm while its concentration in rivers is less than 0.01–0.2 ppm [17]. A small part of Zn in soil, rock, and sediments is constantly removed and transported through the environment by natural erosion processes [18]. Natural phenomena, such as volcanic eruptions, also contribute to the continuous cycling of Zn. Natural emissions of Zn are estimated to reach 5.9 million tons each year [13].

In addition to natural occurrence, industrial activities provide additional sources of Zn contamination. Zn is one of the major pollutants that are released into the environment as a result of industrial activities, mining, smelting, and sewage sludge, as well as persistent use of Zn fertilizers [19]. Zn pollution can be also generated by anthropogenic activities, such as municipal wastewater discharge, coal-burning power plants, manufacturing processes involving zinc, and atmospheric fallout [16,20]. The Zn concentration in soil is closely linked to its different sources. However, its toxicity remains dependent on factors that limit or promote its availability.

In soils, zinc can be present in different forms, mainly as a free ion (Zn^2+^ and ZnOH^+^) or complexed with organic matters. Zn also occurs in the colloidal fraction of the soil coupled with humic compounds, iron and aluminum hydrated oxides or as a constituent of secondary minerals and insoluble complexes [21,22]. The most predominant forms of Zn found in the colloidal fraction are the neutral sulphate ZnSO_4_ and phosphate ZnHPO_4_ [22].

Zn bioavailability is predominantly controlled by adsorption–desorption phenomena and liquid/solid solubility relations [23]. The bioavailability of Zn depends on many factors, namely pH, chemical and mineralogical composition, soil organic matter, root exudates, and rhizospheric microbial communities [8,9,10,23].

Soil pH represents the major factor that influences Zn distribution and speciation in soil because it affects Zn solubility and sorption in the soil solution [24]. Usually, Zn is more bioavailable at an acid pH than at an alkaline pH [25,26]. Zn^2+^ is the predominating form of Zn at a neutral pH (below 7.7) while ZnOH^+^ can be found at pH 7.7–9.1. Above it, the neutral form Zn(OH)_2_ is predominant [22]. Leitenmaier and Küpper, and DalCorso [27,28] demonstrated that an increase in Zn’s bioavailability at a high pH can be explained by a decrease of the intra- and intermolecular hydrogen bonds in humic acid molecules. A small change in the soil pH has a great impact on Zn’s solubility in soil.

The bioavailability of Zn is negatively affected by the presence of phosphatic clay [29] and its coagulation with different mineral structures of the soil, sediment, and water [8]. Cao et al. [30] demonstrated that the presence of phosphate in the soil also affects Zn solubility in water, due to Zn ions’ sorption into the soil mineral compounds. However, Hafeez et al. [31] reported that soils with higher phosphate levels, either from native phosphate or due to the application of phosphate fertilizers, can reduce Zn’s availability. This suggests that phosphate application may be one of the solutions that reduces Zn’s bioavailability in the soils. 

Organic matter plays a key role in governing Zn’s availability in the soil. It improves Zn’s availability by releasing Zn with time and through changes in the physicochemical properties of the soil, which increases its uptake by roots [32]. Clemente et al. [33] found that extractable Zn increases with an elevated organic carbon content in the soil. Organic matter amendment increases Zn’s solubility in soils. Exogenous organic matter releases free Zn into soil solutions, which enables Zn to form complexes with other soil components, and thus change its original solubility and mobilization [34]. However, if the organic matter content in the soil is too high, like in peat and muck soils, this can contribute to a decrease of Zn’s availability due to Zn binding on the solid state of humic substances [31].

It is well known that roots interact with the rhizosphere. Indeed, root activity induces several modifications in the rhizospheric soil proprieties, like the pH, microbial activity, chemical equilibrium, mobility, and bioavailability [33,35]. These changes are induced by root exudates that change the nutrients and metal supplies. Root exudates are released by root cells in the soil, including low molecular weight compounds (amino acids, organic acids, sugars, phenolic compounds) and compounds of high molecular weight (polysaccharides and proteins) [36,37]. Medas et al. [38] reported that root exudates facilitate Zn silicate uptake in *Euphorbia pithyusa* L. In *Arabidopsis halleri*, Tsednee et al. [39] observed that roots release nicotianamine (NA), a main chelator, which has a beneficial effect on improving Zn’s solubilization and thus Zn accumulation. In *Hordeum vulgar, Lolium perenne* L., and different dicots species, roots produce organic molecules that have the ability to form complexes with Zn, thus promoting its mobility [40,41,42].

Rhizospheric microbial communities are an important factor affecting the bioavailability of the soil’s indigenous and exogenous Zn [43]. Among the bacteria, species belonging to the genera *Acinetobacter*, *Bacillus*, *Gluconacetobacter*, and *Pseudomonas* have been described as Zn solubilizers [44]. The mechanisms by which rhizosphere microflora may improve Zn’s solubilization include a reduction of the soil pH, Zn chelation or improvement of root growth. These mechanisms differ from one microorganism to another. Some microorganisms may use one of these mechanisms while others can improve Zn acquisition in plant by using more than one mechanism [26]. Furthermore, the presence of microorganisms in the rhizosphere, in particular mycelium fungi, increases plants’ tolerance to abiotic stress [43]. For example, in *Nocceae praecox*, arbuscular mycorrhizal colonization increased the Zn shoot to root ratio, which reflects better Zn uptake by the root system [45]. 

## 3. Effect of Zn Excess on Plant Development 

Zn is an essential nutriment for all organisms. It is required for the activation of many enzymes in plant cells, such as alcohol dehydrogenase, carbonic anhydrase, and RNA polymerase [4,46]. Zn is also involved in biomembranes’ stabilization by interacting with the phospholipids and sulfhydryl groups of membrane proteins [47]. It can contribute to proteosynthesis, metabolism of carbohydrates, and lipid and nucleic acid synthesis [48,49]. Furthermore, Zn plays a crucial role in oxygen radical production as well as their detoxification. Zn participates in Cu-Zn-SOD enzyme synthesis, a key enzyme involved in the removal of toxic O^−^_2_ radicals, which can be harmful to membrane lipids and proteins [4,47]. Cu-Zn-SOD is essentially localized in chloroplasts; in some plants, it is found in the thylakoid lumen whereas in others it is bound to the thylakoid [50]. At high concentrations, Zn becomes toxic. Jain et al. [51] demonstrated that in *A. thaliana,* treatment with 0.075 mM of Zn did not affect plant development, whereas at concentrations above 0.1 mM, Zn became toxic. 

Zn’s toxic effects depend on its external bioavailable concentration, exposure time, plant genotype, and step of plant development. The most obvious symptoms of Zn toxicity reported in plants are the inhibition of growth, and chlorosis of young leaves (probably the consequence of lower uptake of Fe^2+^ and Fe^3+^), and can lead, in some cases, to cell death [52]. Growth alteration is a consequence of mitosis inhibition. It was reported that excess Zn induces a significant reduction in the mitotic index in *Saccharum* spp and *Triticum aestivum* L, respectively [53,54]. This reduction in mitotic activity could be due to the inhibition of DNA synthesis. 

### 3.1. Effect of Zn Excess on Seed Germination 

Seed is the first phase in the plant life cycle. Studying the effect of excess Zn in seed germination is crucial for understanding Zn’s effects on plant growth and development. The effect of Zn on seed germination depends on the plant species and Zn concentration. It was not significant in *Macrotyloma uniflorum* and in *Pinus sylvestris* at 0.1 mM, but a delay in germination was observed [55,56]. The same results were reported in *Eruca sativa* at 5 mM of Zn, and *Coriandrum sativum* and *Nigella sativa* at 2 mM of Zn. This finding was attributed to the protective effect of seed tegument, which offers protection against metal stress before germination [57,58,59]. However, other studies showed that higher concentrations of Zn considerably reduce the germination of plants, including *Vigna unguiculata*, *Cassia angustifolia*, and *Glycine max* [60,61,62]. Bae et al. [63] observed that the presence of high concentrations of Zn reduced the germination rate in *Trifolium arvense.* Seed teguments seem to play an important role in the protection of seeds against high Zn concentrations.

### 3.2. Effect of Zn Excess on Root Development 

Zn exposure induced significant modifications in the root system architecture [64]. In fact, several studies have revealed that plants exposed to high metal concentrations showed changes in the root morphology and developed higher root branching with a marked curvature and a higher branching percentage in the contact area with the metal [65,66,67,68]. Zn stress also induces a reduction of the primary root length [69]. The repression of root elongation was explained by the inhibition of cell proliferation and subsequent elongation [59]. Li et al. [70] revealed that a reduction of root growth is linked to a significant loss of cell viability in the root tips and to an increased level of lignification in *Triticum aestivum* seedlings exposed to high Zn concentrations. This finding was also reported in *Jatropha curcas* [71] and *Citrus reticulata Blanco* [72]. The observations of a root ultrastructure of *Phyllostachys pubescens* showed that the addition of 0.2 mM Zn had serious effects on root epidermal and root tip cells. Microscopic observations revealed the presence of crystals in the xylem parenchyma, which might obstruct nutrient transport and thus can reduce root growth [73]. In *Senna multijuga* and *Erythrina crista-galli*, a high Zn concentration in the soil induced a linear decrease of the root-specific superficial area, which affects their growth and reduces the capacity of water and nutrient absorption [74]. *Beta vulgaris* L plants grown with a Zn excess are defective in root growth, displaying a brown color with short lateral roots. In this plant, high excess Zn induces a shutdown of the general metabolism in the roots due to a decrease in all of the steps of aerobic respiration [75].

Reactive oxygen species (ROS), such as hydrogen peroxide (H_2_O_2_) and superoxide anion (O_2_^−^), are commonly generated in response to excess Zn in the roots of several plant species [76,77,78]. In *B. napus*, 0.3 mM of Zn induced a strong accumulation of H_2_O_2_ in the roots [79], which can lead to serious cell damage [76,77,78]. However, in the resistant *B. juncea*, ROS levels remained low in the root of Zn-treated plants. This suggests that Zn sensitivity is determined by the level of oxidative species produced in *Brassica* genera [79].

### 3.3. Effect of Zn Excess on Aerial Parts Development 

In the aerial parts of plants, one of the first mechanisms to be affected by zinc toxicity is photosynthesis [80]. With an increasing Zn concentration, photosystem II (PSII) efficiency parameters declined [78]. For example, in *Solanum lycopersicum*, toxic concentrations of Zn (43 ppm) affected plant growth and caused leaf chlorosis, due to the adverse effect of a high Zn concentration on photosynthetic electron transport, loss of plasma membrane integrity, and a decrease of bio-membrane permeability, which result in photosynthesis impairment [81]. The same deterioration was also observed in *Halimione portulacoides*, which is considered as Zn hypertolerant, when cultivated at 70 mmol L^-1^ of Zn [82]. Monnet et al. [83] linked a lack of photosynthetic activity with the production of reactive oxygen species, such as O_2_^−^ or H_2_O_2_, which leads to disassembly of the thylakoids. Azzarello et al. [84] found that antennae pigments’ impairment due to an excessive concentration of Zn may disturb the maximum PSII photochemistry. This decrease in PSII quantum yields was mostly due to a decrease in the basal fluorescence [83]. *Beta vulgaris* L plants grown with excess Zn showed signs of stress, such as their leaf edges rolled inwards. High excess Zn can induce a shutdown of the general metabolism in sugar beet as a result of decreased PSII efficiency [75]. In *Populus* plants, Zn treatment strongly altered the leaf morphology and ultrastructure, inducing a significant thickening of the leaf lamina and spongy tissue [85]. In high concentrations, Zn induces chlorosis and necrosis, and reduces the aerial biomass in *Populus* species and *Brassica rapa* [85,86]. 

Metal-induced stress reduces the rate of photosynthesis and induces reactive oxygen species (ROS) generation [76], which can lead to lipid peroxidation, protein impairment, enzyme inactivation, and DNA damage [77]. Although Zn is a non-redox metal, it generates ROS by indirect mechanisms. Its mechanisms are based on either the stimulation of ROS-producing enzymes, such as Nicotinamide Adenine Dinucleotide Phosphate Hydrogen (NADPH) oxidases, displacing essential cations from specific enzyme-binding sites, or the inhibition of enzyme activities [87]. Under Zn stress, mitogen-activated protein kinase (MAPK) activation results from the activation of oxidative stress in *Oryza sativa* L. MAPKs are serine/threonine kinases involved in the phosphorylation of a number of transcriptional factors [87]. Reactive oxygen species cause the death of plants by damaging membrane lipids, proteins, pigments, and nucleic acids [77,88]. 

Proline accumulation is a widespread process among higher plants in response to zinc and other heavy metal stress. At 1mM of Zn, a rise of proline was mainly observed in the roots of *Triticum aestivum* [70]. The same results were also recorded for *Solanum lycopersicum* tissues with an increase in Zn concentrations (0.05, 0.1, 0.15, and 0.2 mM) [89], and in *Vigna unguiculata* seedlings treated with 0.25 and 0.5 mM of Zn [60]. It is assumed that proline increases the plant’s tolerance to heavy metals through several mechanisms, such as osmoregulation, stabilization of protein synthesis, and enzyme protection against denaturation [88]. Proline accumulation has been shown to alleviate metal-induced oxidative stress by scavenging toxic ROS [90]. Tripathi and Gaur [88] suggested that proline accumulation is somehow triggered by ROS, which allows their direct detoxification without the intervention of antioxidant enzymes. To cope with this damage, cells produce antioxidant enzymes like superoxide dismutase (SOD), peroxidase (POD), catalase (CAT), ascorbate peroxidase (APX), and guaiacol peroxidase [70]. SOD plays an important role in detoxification processes by catalyzing the conversion of free O_2_^−^ to O_2_ and H_2_O_2_ and is associated with stress situations, including zinc stress [79]. In *Myracrodruon urundeuva*, increased SOD activity was seen in Zn-treated plants [91]. In *Plantago major,* CAT enzyme activity increased significantly under Zn stress in the shoot and root [92]. 

## 4. Zn Hyperaccumulator Plants

Despite the toxic effect of heavy metals in plant development, some plant species have developed different strategies to overcome high metal concentrations, which are usually considered phytotoxic in soils. Metal accumulation in the shoots is a naturally selected process that represents one of these strategies [93,94]. Some plants are known for their ability to accumulate abnormally high concentrations of metals, such as Zn, nickel (Ni), manganese (Mn), or lead (Pb), in their aboveground parts to more than 1% of their dry weight [95,96]. These plants are called hyperaccumulators. About 450 plant species have been identified as hyperaccumulators for different heavy metals. Metals are translocated to the shoots and accumulated in the aboveground organs, mainly the leaves. Zn hyperaccumulator plants can accumulate more than 10,000 ppm dry weight in their aerial parts when growing in a natural habitat. For example, *Arabidopsis halleri* and *Noccaea caerulescens* have the ability to accumulate extremely high concentrations of Zn of up to 13,620 and 43,710 ppm of Zn, respectively, when growing in Zn-enriched metalliferous soils [97]. Addititonally, *Dichapetalum subsp. sumatranum* and *D. subsp. pilosum* are strong Zn hyperaccumulators, which can accumulate over 15,660 and 26,360 ppm of Zn in their leaves, respectively [98]. Other researchers have proposed that plants with Zn accumulation levels greater than 3000 ppm should be considered as hyperaccumulators. For example, *Thlaspi ochroleucum*, which can accumulate up to 6300 ppm of Zn, is considered as a physiologically abnormal and hyperaccumulator species [99]. Accordingly, other researchers classify plants based on the shoot:root ratios of metal concentrations and admit that plants with a shoot:root ratio >1 are generally Zn hyperaccumulators while non-accumulator plants’ Zn shoot:root ratios is less than 1 [100]. The literature describes several plants as species that tolerate an environment rich in Zn. 28 Zn-hyperaccumulating plant species have been described to date (Table 1). Most of these species belong to the Brassicaceae family, but other families are also represented, such as Caryophyllaceae and Dichapetalaceae [14,101].

### 4.1. Morphological Response of Zn Hyperaccumulator Plants

Several researchers have reported morphological changes that affect Zn-accumulating plants due to the presence of Zn in the environment. Gallego et al. [105] noticed that in the presence of medium concentrations of Zn, *Noccaea caerulescens* developed better than control plants (grown in normal conditions). Excess Zn affected the growth of *Noccaea caerulescens* plants less. However, there was a significant decrease in the root dry weight at 0.8 mM [106]. Roots can also respond via changes in the growth pattern and morphology. Studies on *S. alfredii* demonstrated that the root length, surface area, and volume visibly increased under 0.5 mM of Zn [107]. Changes in the root morphology of *S. alfredii* were also observed, such as an increase in the diameter class distribution of specific root lengths and specific root surface areas [107]. Many Zn hyperaccumulators (except for trees) have been described with small shallow (<0.5 m) root systems and a high proportion of fine roots that contribute to trace element accumulation [108]. Belouchrani et al. [109] indicated that Zn hyperaccumulation increased the leaf number in *Brassica napus*, changed the root system architecture, and increased lateral root formation after exposure to Zn ions. 

### 4.2. Physiological and Biochemical Responses in Zn Hyperaccumulator Plants

Zn-tolerant plants are characterized by their ability to grow in a Zn-rich medium without showing signs of chlorosis, necrosis, or strong growth inhibition [19]. The maintenance of biomass production even at the highest Zn doses (0.5 mM) indicates the Zn tolerance of *Sedum alfredii* [107]. In *Noccaea caerulescens*, tolerance to Zn is shown by the stability of PSII compared to the control sensitive plants [110].

Hyperaccumulator plants produce root exudates to improve the metal bioavailability. Organic materials, including low molecular weight compounds (amino acids, organic acids, sugars, phenolic compounds) and compounds of high molecular weight (polysaccharides and proteins), mobilize Zn from the soil by acidification and/or chelate secretion [111]. The hyperaccumulator *Sedum alfredii* secretes dissolved organic matter in exudate and has a higher Zn complexation and extraction capacity [112]. Moreover, *S. alfredii* mainly uses these mechanisms to activate metal in the rhizosphere [112]. Furthermore, Dessureault-Rompré et al. [113] suggested that mobile metal-dissolved soil organic matter complexes play an important role in the rapid replacement of available Zn pools in the rhizosphere of hyperaccumulating *N. caerulescens*. Zn might also stimulate citrate production in *N. caerulescens* roots [95]. 

In addition, Tsednee et al. [39] and Leitenmaier and Küpper [27] reported that the secretion of nicotianamine (NA) by the root improves *A. halleri*’s tolerance to Zn. *A. halleri* secretes more NA than *A. thaliana* (non-tolerant). In *A. halleri,* NA synthesis increases when plants are exposed to excess Zn. Xu et al. [114] reported that excess Zn induces an accumulation of nitric oxide (NO) in the roots of the Zn accumulator *Solanum nigrum*. Zn induces the generation of ROS, including hydrogen peroxide (H_2_O_2_) and superoxide radicals. To alleviate the damage caused by ROS production, hyperaccumulator species, like *S. alfredii* and *N. caerulescens*, produce high concentrations of ascorbic acid and glutathione and antioxidant enzymes, such as catalase, peroxidase, and ascorbate peroxidase [115,116].

Metabolite synthesis is also one of the strategies used by hyperaccumulator plants to prevent toxicity from heavy metals [117]. Glutathione (GSH) amounts increased in a metallicolous population of *S. alfredii* in comparison with the non-metallicolous one and might serve as an antioxidant or metal chelator involved in Zn detoxification [118]. Instead of GSH, phytochelatins (PCs), small cystein-rich peptides, do not have a specific role in the binding of Zn in hyperaccumulator plant species [95]. As reported in a metallicolous population of *Sedum alfredii*, PCs and cysteine (Cys) were not detected in any tissue [118]. It was previously revealed that an important PC’s synthesis in heavy metal-exposed plants would cost a considerable amount of energy required for the sulphate reduction [117]. Furthermore, metallothioneins (MTs) are reported to be involved in Zn hyperaccumulation. The Zn hyperaccumulator *N. caerulescens* displays constitutively higher MT expression than *A. thaliana*. Moreover, NcMT1 and NcMT2 showed overexpression patterns in *N. caerulescens* organs and in response to Zn exposure, which suggests a possible role of MTs in the tZn hyperaccumulation process [119]. 

## 5. Molecular Mechanisms of Zn Hyperaccumulation

After bioactivation in the rhizosphere, Zn is then absorbed by the roots through mass flow and diffusion mechanisms. In plants, Zn can follow two different pathways to reach the xylem: (i) Symplastic route: The cytoplasm of neighboring cells in the root tissues is connected by cytoplasmic bridges (plasmodesmata) in the cell wall, forming a symplastic continuum without membrane barriers. Cytoplasmic Zn then follows this path to the pericycle without spending energy [100]; and (ii) the apoplastic route, where Zn moves across the cell wall and intercellular spaces. Zn is then blocked in the endodermis by lamellae deposition in the casparian strip [100]. An unusual root feature (cells of the perendodermis with irregularly thickened tangential walls with deposition of lignin) was observed in the hyperaccumulator *N. caerulescens* compared to the non-hyperaccumulator *N. arvense* [120]. van de Mortel et al. [121] suggested that the plant uses lignin depositions to control metal influx or to prevent excess metal efflux from the central stele, providing the plant with the capacity to maintain a lower metal concentration in the cortex and then busting the metal uptake from the soil. When reaching the endodermis level, Zn must move in a symplastic way using transporters. In this way, Zn ions flux to the stele can be reduced through vacuolar retention [122]. Nevertheless, less Zn vacuolar sequestration in the root is an important mechanism for Zn hyperaccumulation. It was found that *N. caerulescens* stored approximately 2.5 times less Zn in the root cell vacuoles compared to the sensitive species *N. arvense* [122]. Additionally, the main sites of Zn accumulation in *P. griffithii* were the apoplast, particularly in the cell walls [123]. 

In general, the molecular mechanisms involved in metal hyperaccumulation are essentially derived from the mechanisms involved in metal homeostasis in plants. Several studies showed that the same genes involved in metal homeostasis are differentially expressed in hyperaccumulators compared to related non-accumulator species as a result of gene duplication and/or changes in promoter regulation [124,125]. In the case of Zn, genes involved in the hyperaccumulation strategy were mostly identified in both *A. halleri* and *N. caerulescens* [126,127]. 

### 5.1. Zinc Uptake

The rhizosphere is the first area of contact between metal and plants. In the soil, Zn occurs preferably in a crystalline form in iron-magnesium minerals, such as sulfide (ZnS). Zn is largely adsorbed in changeable forms, mainly as Zn^2+^, ZnOH^+^, and ZnCl^+^, onto clay surfaces and organic matter [11]. Roots generally absorb zinc as Zn^2+^ ions except at high pH, where it is absorbed as ZnOH [128].

**Figure 1:**
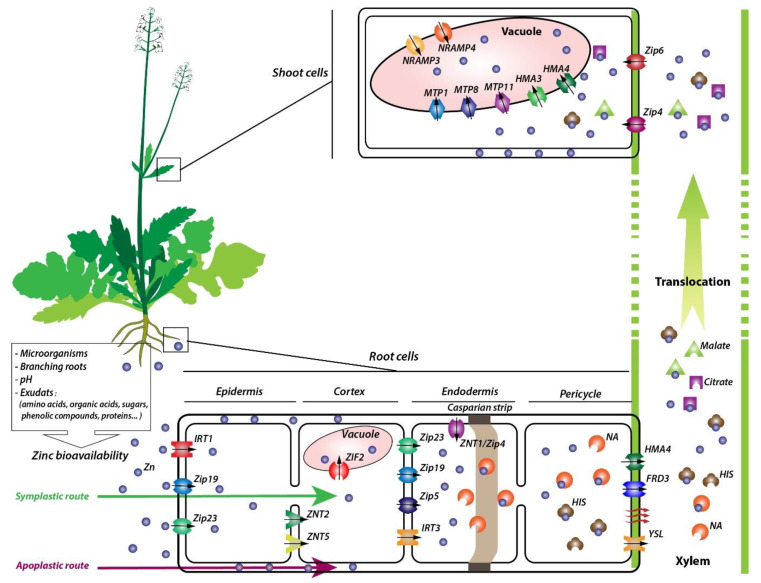
A model of the mechanisms that occur in hyperaccumulation plants upon exposure to zinc (Zn): Zn ion uptake, chelation, transport, and sequestration. Zn bioavailability can be influenced by several factors, such as microorganisms, branching roots, pH, and exudates. Once adsorbed by the roots, Zn can be absorbed by an apoplastic route: A passive diffusion through cells, or by a symplastic route via transporters. Within the latter path, Zn absorption by epidermis cells is mainly promoted by IRT1, ZIP19, and ZIP23. To reach the cortex, Zn can be directly diffused or by means of ZNT2 and ZNT5. Then, Zn can either be stocked in vacuoles (promoted by ZIF2) or transported to the endodermis through the following transporters: ZIP23, ZIP19, ZIP5, and IRT3. Zn following the apoplastic route is stopped by the casparian strip, and then enters the endodermis via ZNT1/ZIP4. At this level, Zn can be chelated by nicotianamine (NA) or directly diffused to pericycle cells where a part can also be associated to histidine (His). The unchelated Zn can reach the xylem through direct diffusion or via YSL, ferric reductase defective 3 (FRD3,) and HMA4. Zn then crosses the xylem as a Zn-free form or coupled with His, citrate, or malate. To enter the leaf cells, Zn can passively penetrate in chelated forms or as the Zn-free form via ZIP4 and ZIP6 proteins. It is then sequestrated inside the vacuole through MTP1 (metal tolerance proteins 1), MTP8, MTP11, NRAMP3, NRAMP4, HMA3, and HMA4 transporters, or blocked in the cell wall.

Many transporters belonging to the zinc-regulated transporter/iron-regulated transporter-like proteins (ZRT-IRT-like protein or ZIP family) and involved in Zn radial transport to the stele in roots have been identified. In *N. caerulescens*, *Nc*ZNT1, a major contributor to Zn transport across the endodermal cell, was isolated [121]. Constitutive overexpression of *NcZNT1,* a plasma membrane-located metal transporter, in *A. thaliana* enhanced the tolerance to excess Zn exposure and increased the accumulation of Zn compared to wild-type plants [129]. Furthermore, *Nc*ZNT 2 and 5, respectively orthologs of *A. thaliana* iron-regulated transporter 3 (IRT3) and ZIP5, are involved in Zn transport in the roots [121,130]. Additionally, *IRT3* was detected in the plasma membrane of *A. halleri* cells in the roots after exposure to Zn [129,131,132]. Interestingly, high levels of *IRT3* expression were found in the roots of Zn-hyperaccumulating species, such as *A. halleri* and *N. caerulescens* [11,131]. Other studies conducted by Shanmugan et al. [133] showed that IRT1 located in the plasma membrane is involved in Zn transport. Lin et al. [129] also observed a subcellular location of this transporter in the plasma membrane, which confirms its importance in the passage of these ions to the cellular cytoplasm. *ZIP19* and *ZIP23* expression was significantly induced in *A. halleri* and *N. caerulescens* roots in the presence of Zn [129]. In *A. thaliana*, high levels of *ZIP19* and *ZIP23* expression were observed in a Zn deficient culture medium [134], which suggests that the upregulation of these two genes in *A. halleri* and *N. caerulescens* might be correlated with a low Zn content in the rhizosphere due to the high activity of heavy metal ATPase 4 (HMA4) (involved in Zn translocation from the xylem to aboveground parts) [124,129] (Figure 1).

In addition to the ZIP family, another gene is described to have a role in zinc uptake. In *Arabidopsis*, Remy et al. [135] showed that ZIF2 (zinc-induced facilitator 2) is primarily localized at the tonoplast of root cortical cells and mediates Zn uptake (Figure 1).

### 5.2. Zn Xylem Loading and Transport Processes 

Once excess Zn reaches the endoderm of hyperaccumulator plants, Zn is loaded with root vessels to xylem vessels [11]. In the xylem parenchyma, Zn will be chelated with low molecular weight ligands to prevent its retention by surrounding cell walls [11,136]. For instance, Zn translocation in *A. halleri* is accompanied with strong nicotianamine (NA) secretion, a non-proteinogenic amino acid that, once bound to Zn, prevents its rapid absorption [137,138]. The nicotianamine synthase (NAS) catalyzes NA synthesis from three molecules of S-adenosyl-methionine [139]. *NAS1*, *NAS2*, and *NAS3* genes expression revealed a difference between hyperaccumulator and non-hyperaccumulator plants. In addition, expression studies have showed that in *N. caerulescens,* NA synthesis is governed by NAS2 while in *A. halleri*, it is controlled by NAS2 and NAS3 [121,140]. Inhibition of NA synthesis in the root induces a decrease in Zn translocation. After an analysis of xylem exudates, Cornu et al. [141] suggested that organic acids, such as malate and citrate, are the main ligands in xylem, while NA controls the loading rate of xylem only. For histidine (His), even if its specific mechanism of action is unclear, the supply of exogenous histidine contributes to the transport of Zn and increases the xylem loading process [142]. Milner and Kochian [143] found that Zn was complexed with His in the root but becomes free hydrated Zn^2+^ in the xylem whose small amount is bound with organic acids (Figure 1).

Zn translocation from the roots to the shoots involves the action of proteins belonging to the heavy metal ATPase “HMA” family. In *A. halleri*, heavy metal ATPase 4 (HMA4) and 2 (HMA2) act as a key component contributing to Zn hyperaccumulation and hypertolerance [144,145,146]. With a plasma membrane localization in the root pericycle cells, they promote Zn^2+^ efflux from these cells and are associated with its loading in the root xylem [11,147]. Transcriptional-level analysis in hyperaccumulator plants revealed a strong induction of these genes. *HMA4* expression was 4 to 10-fold higher in roots and at least 30-fold higher in shoots of *H. halleri* compared to *A. thaliana* [131]. The increased expression of *HMA4* is a result of tandem triplication and cis-regulatory changes that can activate the promoters of all three *HMA4* copies [145]. Some genes from the ZIP family, likely *ZIP4* and *IRT3*, are also involved in root-to-shoot translocation. It has previously been shown that the overexpression of *AtIRT3* in *A. thaliana* increased Zn accumulation in the shoots, suggesting a potential involvement of this gene in Zn loading for long-distance transport [148] (Figure 1).

Other genes involved in Zn transfer are those of the yellow stripe-like (YSL) family. YSL transporters have been implicated in xylem loading and unloading of Zn [149]. YSL proteins are involved in Mn, Zn, Cu, Ni, Cd, as well as Fe transport. They mainly mediate the cellular uptake of metals that are complexed to non-proteinogenic amino acids [150]. Similar to YSL, FRD3 (ferric reductase defective 3), a MATE (multidrug and toxin efflux) transporter, has been described as an essential regulator of Zn excess [151]. *FRD3* expression was exclusively detected in the pericycle and cells surrounding the vascular tissues [152]. This protein could be of primary importance in the distribution of metals between cells [136] (Figure 1). FRD3 is essential for Fe/Zn translocation via the xylem [153,154]. 

In *A. thaliana*, there is another important efflux transporter: Plant cadmium resistance 2 (PCR2). PCR2 functions as a Zn efflux transporter, which contributes to Zn translocation and detoxification [155]. However, its implication in Zn hyperaccumulation is not understood.

After Zn loading in the xylem using different transporters from three major families HMA, ZIP, and YLS, Zn reaches the leaves, where it is mainly bound to organic acids, such as malate and citrate, and then sequestered in vacuoles.

### 5.3. Zinc Sequestration in the Aboveground Part of the Plants

After been chelated (with NA, His, etc.) in the roots and translocated towards the xylem, Zn reaches the shoot, where it is stored bound to chelators (organic acids). A small portion of Zn can also be found as free ions or bound to the cell wall or histidine [143]. In *N. caerulescens* shoots, concentrations of malate, oxalate, succinate, and citrate are detected only after high Zn exposure [95]. 38%, 9%, 16%, and 12% of the total Zn was associated with citrate, oxalate, His, and the cell wall, respectively, while 26% remained as free Zn^2+^ ions in the shoots of *N. caerulescens* [95]. Afterward, chelated Zn is stored in different cells/tissues depending on the plant species. For example, *A. halleri* promotes the accumulation of Zn at the base of trichomes and also in mesophyll cells [156] whereas *N. caerulescens* stores metals in the leaves’ epidermis [156]. In *Sedum alfredii*, Zn is at the level of the epidermis of the stems and leaves [157]. In contrast, mesophyll cells accumulate small amounts of Zn in hyperaccumulator plants as an adaptation strategy [124,158]. Zn accumulation includes efficient metal import and loading in the vacuoles along with a regulated redistribution from this compartment. The potential actors involved in these mechanisms comprise members of several transporter families.

Yang et al. [159] identified the *SaZIP4* gene as a key Zn transporter in the Zn hyperaccumulation process of *Sedum alfredii*. A subcellular localization analysis showed that *ZIP4* is located in the plasma membrane and is involved in Zn uptake in the shoots but also in the roots. Another member of the same family, ZIP6, located in the plasma membrane of the shoot, promotes Zn uptake as its expression was predominant in the shoots of *A. halleri* [100,160] (Figure 1). 

Once in the cytosol, Zn is stored in the vacuoles as a strategy of hyperaccumulator species to prevent Zn damage of vital organelles, such as chloroplast. Sitko et al. [161] showed that metallicolous populations of *A. halleri* were characterized by a high chlorophyll content compared to non-tolerant plants. This finding can be explained by the inhibition of Zn accumulation in chloroplasts in this species [161]. It was confirmed that HMA1, a member of the P1B-type ATPase superfamily, was identified and localized on the chloroplast envelope in *A. thaliana* [162] (Figure 1). Furthermore, *Arabidopsis HMA1* knockout plants were more sensitive to Zn and accumulated a high amount of Zn in the chloroplast compared to wild-type plants [162]. These authors showed that HMA1 is associated with Zn^2+^ transport from the chloroplasts to the cytoplasm under conditions of high levels of zinc, avoiding damage to the organelle. However, the implication of HMA1 in Zn transport in hyperaccumulator species has not been confirmed yet. We can thus suggest the possible involvement of HMA1 in the inhibition of Zn accumulation in the chloroplasts of hyperaccumulator species. 

It was confirmed that members of the MTP/ABCC (metal tolerance proteins/ATP binding cassette C), belonging to the superfamily of the cation diffusion facilitator (CDF) protein, are involved in Zn loading in the vacuoles [163]. In the leaves, the vacuolar protein MTP1 plays a key role in Zn sequestration and detoxification. It was reported that MTP1 contributes to Zn accumulation in the shoots of *A. halleri* [163] Likewise, in *N goesingense*, TgMTP1 protein is accumulated in high levels in the vacuolar membrane in the shoot, which confirms its implication in Zn tolerance and accumulation enhancement in this species [164]. Next to MTP1, upregulation by high Zn of *MTP8* and *MTP11* was also confirmed in the shoots of the Zn hyperaccumulators *N. caerulescens* and *A. halleri* [126]. Like MTP1, ZNT5 is also an important player in the sequestration of Zn into the epidermal storage cells [106,165] (Figure 1).

Recently, HMA4, previously described as a Zn xylem transporter, and HMA3, another member of the P1B-type ATPase superfamily, were studied regarding their implication in Zn/Cd sequestration in shoots [156]. The regulation of both *HMA3* and *HMA4* was analyzed at the tissue and cellular level in the Zn hyperaccumulators *A. halleri* and *N. caerulescens*. In *A. halleri*, the highest expression of *HMA3* and *HMA4* was found in the mesophyll, while in *N. caerulescens, HMA3*, and *HMA4* were significantly upregulated in the bundle sheath of the vein. This can be explained by the final storage sites; the epidermis for *N. caerulescens* and mesophyll for *A. halleri* [156]. HMA3, located in the vacuolar membrane, participates in vacuolar sequestration of Zn [156]. In addition to these genes, four other genes belonging to the NRAMP (natural resistance-associated macrophage protein) family, *NRAMP1*, *NRAMP3*, *NRAMP4*, and *NRAMP5,* have been described to be involved in Zn sequestration in the vacuole of leaf cells. These transporters use the transmembrane proton gradient to facilitate the transport of a broad range of divalent cations toward the cytosol [166]. In *N. caerulescens, NRAMP1* and *NRAMP5* were found to be highly expressed in the shoots specifically [167,168]. High expression of the *NRAMP3* gene was observed in the leaves of the Zn hyperaccumulating *A. halleri* [168]. *NcNRAMP4* was implicated in Zn hypertolerance with localization at the vacuolar membrane [169], but the precise role *NcNRAMP3* is yet to be determined.

## 6. Genetic Basis of Zn Hyperaccumulation 

Most research on Zn hyperaccumulators has focused on the physiological and molecular mechanisms of Zn uptake, transport, and sequestration, but relatively little is known regarding the genomic variation and genetic evolution of the Zn hyperaccumulation characteristic. 

It is known that interspecific heavy metal tolerance can occur differently. For example, *A. halleri* is geographically distributed in Europe and eastern Asia and has been divided into metallicolous, non-metallicolous, and hybrid populations. Comparative studies have shown that metallicolous populations accumulate more Zn than non-metallicolous populations [94]. These observations were contradicted by those of Frérot et al. and Stein et al. [144,170]. It was suggested that the hyperaccumulation characteristic in *A. halleri* is due to population evolution rather than a new mutation [171]. This same population variation was also observed in *N. caerulescens* [172]. Meanwhile, Nowak et al. [173] described of a reduction in the hyperaccumulation capacities between populations. There are no known cases of major genetic polymorphisms in which some members of a species are capable of hyperaccumulation and others are not. Whereas, Lin and Aarts [174] described a similar Zn absorbance between the two populations of *Sedum alfredii*. Similarly, biomass changes depend on the population, for example, an excess of Zn in the medium induces the growth of *S. alfredii* from metallicolous sites whereas for non-metallicolous populations, it causes a decrease in the biomass accompanied with several toxic symptoms [159]. 

The random amplified polymorphic DNA (RAPD) method was used to compare the genomic variation of *Sedum* hyperaccumulating ecotype (HE) and non-hyperaccumulating ecotype (NHE), and revealed that the Zn/Cd hyperaccumulation trait was related to SH-containing compounds [175]. Recently, Yang et al. [176] identified molecular markers single nucleotide polymorphisms (SNPs) and simple sequence repeats (SSRs) of *S. alfredii*, which constitutes an important resource for genome mapping to identify hyperaccumulation and hypertolerance traits. From this approach, 18 divergent orthologous genes involved in the transcription and translation processes, protein metabolism process, Ca^2+^ signaling pathway, stress response process, and signal transduction process were identified in HE [176]. Schvartzman et al. [94] examined the mechanisms underlying the intraspecific variation in two geographically distant metallicolous populations of *A. halleri*. The transcriptomic analysis revealed that the geographic origin represented a major source of variation in the accumulation properties and gene expression profiles. This study suggested that the two metallicolous populations developed different strategies to adapt to an increasing Zn content in the soil through Zn crosstalk modifications [94]. 

The revelation of a genetic basis of hyperaccumulation traits can be achieved by cross-referencing between tolerant and sensitive species. A study performed in *N. caerulescens* showed the presence of three quantitative trait loci (QTLs) related to Zn accumulation [177]. QTL mapping of zinc tolerance was used to analyze the first-generation backcross progeny from *A. halleri* ssp. and its non-tolerant relative *A. lyrata* ssp. *petraea* [178]. The authors identified three QTLs and showed that in all these QTLs, Zn tolerance was improved by *A. halleri* alleles. This result was explained by the positive selection of higher Zn tolerance in this species [178]. Whether it is an adaptation or a reduction in this capacity, differences between populations remain a very important point to study in order to understand the evolutionary steps that lead to zinc tolerance and/or accumulation. 

## 7. Conclusions

The understanding of Zn uptake and hyperaccumulation mechanisms in plants has been considerably advanced in recent years. Some plants have developed a range of physiological adaptation strategies to tolerate and hyperaccumulate Zn in their aboveground parts. Many genes belonging to different families (*HMA*, *ZIP*, *YSL*, *MTP*, etc.) have been identified as being involved in Zn tolerance and hyperaccumulation, and transcriptomic analysis of contrasting populations has clarified the evolutionary mechanisms of the Zn hyperaccumulation characteristic. 

All these advances are of high relevance for understanding the physiological processes of Zn transfer from the soil to the plant’s tissues and cellular compartments. Hence, the application of Zn hyperaccumulator plants for phytoremediation and clean-up of Zn-contaminated soils has become a reality. Phytoremediation is a green, eco-friendly, and low-cost technology recognized as an effective method for the remediation of metal-contaminated soils [179]. However, exploitation of hyperaccumulation for soil decontamination is limited due to the low biomass and slow growth of hyperaccumulator plants and the low bioavailability of Zn in the soil [111,180]. Rhizosphere microbes, especially the PGPR, endophytic bacteria, and mycorrhizae, and the application of fertilizers and exogenous chelators can mend Zn mobility and bioavailability and effectively assist in phytoremediation [181]. Moreover, recent advances in genetic engineering and genome editing tools, like clustered regularly interspaced palindromic repeats (CRISPR) strategies [182], will allow a better understanding of the molecular mechanisms involved in Zn uptake, transport, and accumulation in plants. This will lead to improvements in Zn hyperaccumulation and can open new possibilities for soil decontamination. 

## Figures and Tables

**Table 1:** List of Zn hyperaccumulator plants.

Species	Family	Hyperaccumulation Criteria	References
*Justicia procumbens*	Acanthaceae	>10,000 ppm in LDW	[101]
*Arabidopsis helleri*	Brassicaceaa	>10,000 ppm in LDW	[97]
*Noccaea caerulescens*	Brassicaceaa	>10,000 ppm in LDW	[97]
*Arabis paniculata*	Brassicaceae	>10,000 ppm in LDW	[102]
*Noccaea eburneosa*	Brassicaceae	Zn concentration in shoot %DW 1.05	[103]
*Noccaea alpestre*	Brassicaceae	>10,000 ppm in LDW	[103]
*Noccaea bulbosum Spruner*	Brassicaceae	Zn concentration in shoot %DW 1.05	[103]
*Noccaea calaminare*	Brassicaceae	>10,000 ppm in LDW	[103]
*Noccaea limosellifolium*	Brassicaceae	Zn concentration in shoot %DW 1.10	[103]
*Noccaea praecox*	Brassicaceae	>10,000 ppm in LDW	[103]
*Arabis gemmifera*	Brassicaceae	>10,000 ppm in LDW	[101]
*Noccaea* *goesingense*	Brassicaceae	>10,000 ppm in LDW	[101]
*Noccaea brachypetalum*	Brassicaceae	Zn concentration in shoot %DW 1.53	[101]
*Noccaea cepaeifolium* subsp *Rotundifolium*,	Brassicaceae	Zn concentration in shoot %DW 2.10	[101]
*Noccaea stenopterum*	Brassicaceae	>10,000 ppm in LDW	[101]
*Noccaea tatrense*	Brassicaceae	>10,000 ppm in LDW	[101]
*Minuartia verna*	Caryophyllaceae	Zn concentration in shoot %DW 1.14	[101]
*Polycarpaea synandra*	Caryophyllaceae	>3000 ppm (6960 ppm DW)	[101]
*Sedum alfredii*	Crassulaceae	shoot: root ratio >1	[101]
*Sedum plumbizincicola*	Crassulaceae	>10,000 ppm in LDW	[101]
*Dichapetalum geloniodes subsp.sumatranum*	Dichapetalaceae	>10,000 ppm in LDW	[101]
*Dichapetalum gelonioides*	Dichapetalaceae	>10,000 ppm in LDW	[101]
*Anthyllis vulneraria*	Fabaceae	>10,000 ppm in LDW	[101]
*Haumaniastrum katanngense*	Lamiaceae	Zn concentration in shoot %DW 1.98	[103]
*Ficus parietalis*	Moraceae	-	[101]
*Potentilla griffithii*	Rosaceae	>10,000 ppm in LDW	[104]
*Rinorea longiracemosa*	Violaceae	-	[101]
*Viola calaminaria*	Violaceae	>10,000 ppm in LDW	[103]

LDW: Leaf Dry Weight; DW: Dry Weight.
